# An Update on Sphingolipidomics: Is Something Still Missing? Some Considerations on the Analysis of Complex Sphingolipids and Free-Sphingoid Bases in Plasma and Red Blood Cells

**DOI:** 10.3390/metabo12050450

**Published:** 2022-05-17

**Authors:** Camillo Morano, Aida Zulueta, Anna Caretti, Gabriella Roda, Rita Paroni, Michele Dei Cas

**Affiliations:** 1 Department of Pharmaceutical Sciences, Università degli Studi di Milano, 20133 Milan, Italy; camillo.morano@unimi.it (C.M.); gabriella.roda@unimi.it (G.R.); 2 Neurorehabilitation Department, IRCCS Istituti Clinici Scientifici Maugeri di Milano, 20138 Milan, Italy; aida.zuluetamorales@icsmaugeri.it; 3 Department of Health Sciences, Università degli Studi di Milano, 20142 Milan, Italy; anna.caretti@unimi.it (A.C.); rita.paroni@unimi.it (R.P.)

**Keywords:** sphingolipids, sphingolipidomics, sphingoid bases, lipidomics, mass spectrometry

## Abstract

The main concerns in targeted “*sphingolipidomics*” are the extraction and proper handling of biological samples to avoid interferences and achieve a quantitative yield well representing all the sphingolipids in the matrix. Our work aimed to compare different pre-analytical procedures and to evaluate a derivatization step for sphingoid bases quantification, to avoid interferences and improve sensitivity. We tested four protocols for the extraction of sphingolipids from human plasma, at different temperatures and durations, and two derivatization procedures for the conversion of sphingoid bases into phenylthiourea derivatives. Different columns and LC-MS/MS chromatographic conditions were also tested. The protocol that worked better for sphingolipids analysis involved a single-phase extraction in methanol/chloroform mixture (2:1, *v*/*v*) for 1 h at 38 °C, followed by a 2 h alkaline methanolysis at 38 °C, for the suppression of phospholipids signals. The derivatization of sphingoid bases promotes the sensibility of non-phosphorylated species but we proved that it is not superior to a careful choice of the appropriate column and a full-length elution gradient. Our procedure was eventually validated by analyzing plasma and erythrocyte samples of 20 volunteers. While both extraction and methanolysis are pivotal steps, our final consideration is to analyze sphingolipids and sphingoid bases under different chromatographic conditions, minding the interferences.

## 1. Introduction

Sphingolipids are a ubiquitous class of lipids, whose structure always comprises a long-chain base, usually sphingosine (Sph) or sphinganine. Their name derives from the mythological figure of the sphynx, because of their enigmatic nature [1]. Sphingolipids are commonly divided into two major classes: ceramides (Cer) and complex sphingolipids. Cer are “de novo” biologically synthesized by attaching a fatty acid to the amine group of dihydrosphingosine (dhSph) through an amidic bond, and are mostly found in the outer leaflet of the plasma membrane. Cer are then catabolized to Sph and sphingosine-1P (S1P) which will exit the pathway by degradation to palmitoyl aldehyde and phosphoethanolamine. Complex sphingolipids, on the other hand, comprise many different subclasses, such as sphingomyelins (SM), made up of a polar head such as choline or serine, and glycosphingolipids, which are, in turn, classified according to the number of sugar residues attached to the carbon chain [2,3]. Other than their role in the formation and modulation of biological membranes, sphingolipids, especially Cer, the “central hub” of sphingolipids metabolism, and S1P, are believed to be responsible for many different signaling functions in the organism such as apoptosis, inflammation, cell proliferation, and differentiation.

Due to the various roles that sphingolipids have, any alteration of their metabolism could be part of pathological mechanisms or, sometimes, could be the reason for the diseases themselves [4,5,6,7,8]. The analysis of the whole set of sphingolipids in a biological system is referred to as “*sphingolipidomics*”, and is now standardly carried out through liquid chromatography coupled to mass spectrometry to characterize and differentiate simultaneously the numerous species of sphingolipids belonging to the different subclasses [9,10]. However, due to the high variety of chemical structures, one of the main issues remains the extraction and proper handling of samples to achieve a yield that could well represent the actual concentrations of all sphingolipids in the system under analysis as already postulated in untargeted lipidomics [11]. In fact, on one hand, Cer and complex sphingolipids can be easily characterized using a solvent extraction followed by alkaline methanolysis [12,13], which remains the method of choice for sample handling; on the other, free sphingoid bases are hard to extract and their analysis can be quite challenging. Indeed, while there is a growing general interest in achieving a common protocol for the analysis of free sphingoid bases such as Sph and S1P, as they appear to be, as mentioned, important biological mediators, they are fairly difficult to be detected using LC-MS/MS. The reason for this is double-fold: (1) short liquid chromatography does not allow one to properly separate free sphingoid bases from any interferent in the system; and (2) their chemical nature makes it difficult to obtain proper ionization of the compounds.

Our work aimed to compare different methods of sample handling and extraction for sphingolipids. Furthermore, we evaluated whether a derivatization step by phenylisothiocyanate (PITC) could improve the detection and analysis of free sphingoid bases.

## 2. Results and Discussion

### 2.1. Set Up of the Extraction Procedure

**Figure 1:**
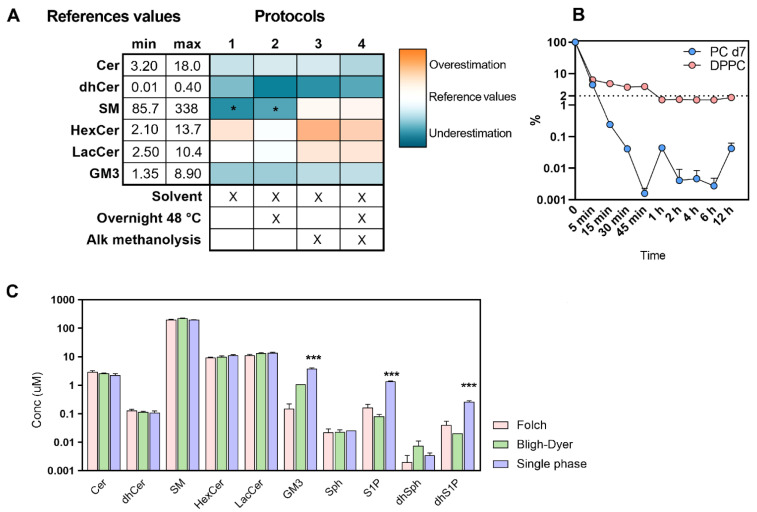
(**A**) Quantification of sphingolipids in a plasma pool from healthy volunteers (*n* = 20) as a function of different extraction protocols and comparison to the reference values found in the scientific literature. On the left of the heatmap, the range of concentration (µM) of sphingolipids in plasma EDTA from healthy volunteers found in the scientific literature [19,20,21,22,23,24,25,26,27]. For visualization, data were scaled to reference values and reported as a fold-change logarithm. Those significantly modulated were evaluated by performing repeated measures one-way ANOVA and the Dunnett post hoc test. The different steps in each protocol are schematized under the heatmap and their occurrence is marked with an “X”. (**B**) Estimation of plasma phospholipids content after alkaline hydrolysis (KOH 73 mM) over time (0, 1, 2, 4, 6, 12 h) at 38 °C. Data were visualized as a percentage of DPPC and PC d7 with respect to untreated samples. Each point represents the mean of *n* = 2 technical replicates. (**C**) Comparison of the traditional liquid–liquid extraction for total lipid content (Folch *n* = 3 and Bligh-Dyer *n* = 3) and the single-phase extraction (*n* = 3) for the recovery of sphingolipids in a plasma pool from healthy volunteers (*n* = 20). Statistical differences were measured by one-way ANOVA and the Dunnett post hoc test against monophasic extraction. *p* values are schematized as follows: * < 0.05; *** < 0.001.

We tested four different extraction protocols (Materials and Methods) to evaluate whether different conditions could deeply affect the recovery of sphingolipids. As displayed in Figure 1A, the more complex classes of sphingolipids do not seem to be impacted using the four different procedures, except SM, which appears to be underestimated using the first two protocols. Alkaline methanolysis is useful to disrupt the ester bond in phospholipids while maintaining the amide linkage unaltered, which is characteristic of sphingolipids. Especially using low-resolution triple quadrupole, the need for distinguishing or chromatographically separating phosphatidylcholine (PC) and SM is factual since they can co-elute and/or overlap in mass transition (e.g., SM 38:3 m/z 771.6115 > 184 and PC O-36:2 m/z 772.6209 > 184; SM 42:4 m/z 809.6494 > 184 and PC 38:4 m/z 810.6004 > 184) competing irremediably in their quantification [14]. In every condition (Figure 1B) here reported, incubation at 38 °C from 1 h to 12 h can effectively reduce the plasma physiological phospholipids content of about 98.5% (estimated on dipalmitoylphosphatidylcholine, DPPC) and more than 99.9% on added deuterated internal standard (phosphatidylcholine (15:0–18:1) d7, PC d7). By contrast, a shorter time (inferior to 1 h) allows a lower reduction of phospholipids, which can be estimated between 93 and 95% with respect to not-treated samples. The warm incubation overnight (48 °C) has been historically introduced to uniformly level the lipid in the extracting solvent since different sphingolipids can have high phase transition temperatures. However, we believe that this passage could be shortened since its beneficial effect was not observed (see below) [12]. The traditional liquid–liquid extraction protocols firstly proposed by Folch and Bligh-Dyer [15,16] and the monophasic extraction—here and elsewhere described [13,17,18]—are essentially identical in extraction rate for the content of Cer, dihydroceramides (dhCer), SM and glycosphingolipids (Figure 1C). The prominent polarity of acidic glycosphingolipids—such as the simplest gangliosides (GM3)—does not grant a standardized recovery in the bottom chloroform phase of Folch (mean ± SD, 0.15 ± 0.07 vs. 3.8 ± 0.21 single-phase) and also in the more polar Bligh-Dyer (1.12 ± 0.03 vs. 3.8 ± 0.21 single-phase) protocols. The use of Folch and Bligh-Dyer also emphasizes the recovery of the sphingoid bases especially in their phosphate forms S1P and dihydrosphingosine-1-phosphate (dhS1P). This effect was also noticeable on the internal standard used for this purpose (sphinganine d17:0) whose extraction fate is diminished in Folch by 55% and in Bligh-Dyer by 26% with respect to monophasic extraction.

**Figure 2:**
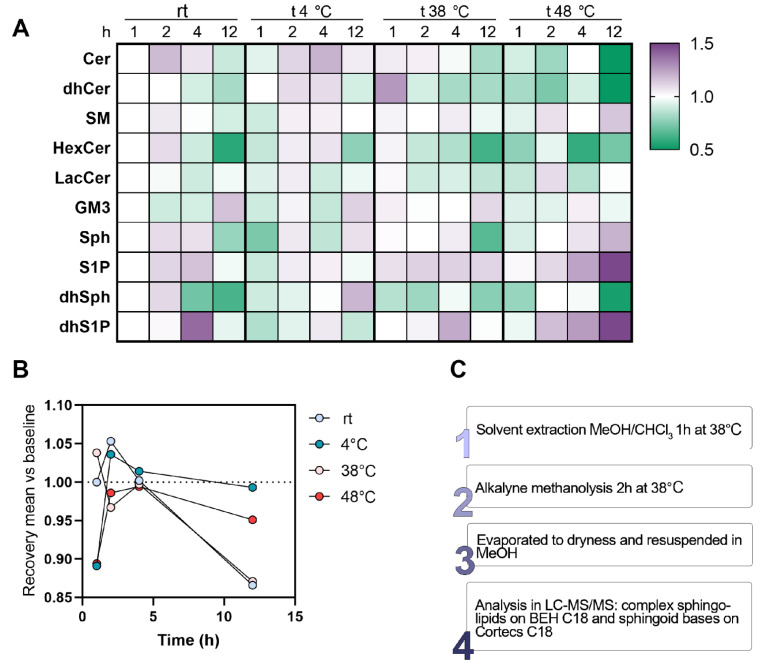
(**A**) Effects of time (1/2/4/12 h) and temperature (rt/4/38/48 °C) on the recovery of sphingolipids from plasma using a single-phase extraction (*n* = 2 per each condition). Data were scaled for visualization on the recovery obtained for 1 h at rt (22 °C). (**B**) The effects of times (1/2/4/12 h) and temperatures (room temperature, rt/4/38/48 °C) on the recovery of sphingolipids from plasma. Data were scaled for visualization on the recovery obtained for 1 h at rt (22 °C, baseline). (**C**) Scheme of the final steps included in the protocol.

The effects of times (1/2/4/12 h) and temperatures (room temperature, rt/4/38/48 °C) on the recovery of sphingolipids from plasma were also considered and the results are graphed in Figure 2A. As already postulated above, the overnight extraction (12 h) seems to be futile or even counterproductive, thus we believe that this passage could be shortened between 1 and 2 h. The incubation at 48 °C is overall worthless and detrimental, especially on complex sphingolipids (e.g., Cer, dhCer and hexosylceramides, HexCer). The only species which strongly benefit from this long and hot period of extraction are phosphate forms of sphingoid bases (+48% at 48°; +25% at 38° vs. baseline condition 1 h at rt). We demonstrated that the recovery of either: (a) 1 h at 38 °C; (b) 2 h at rt; or (c) 2 h at 4 °C is essentially superior and interchangeable between them since their mean recovery is +5% (Figure 2B) in respect to baseline (1 h at rt). The extraction with a temperature between 38 and 48 °C and prolonged from 2 to 4 h revealed a slightly decrease in the recovery of plasma sphingolipids. The results presented in this paragraph are summed up in a final protocol proposed and outlined in Figure 2C.

### 2.2. Choosing the Best Analytical Condition

**Figure 3:**
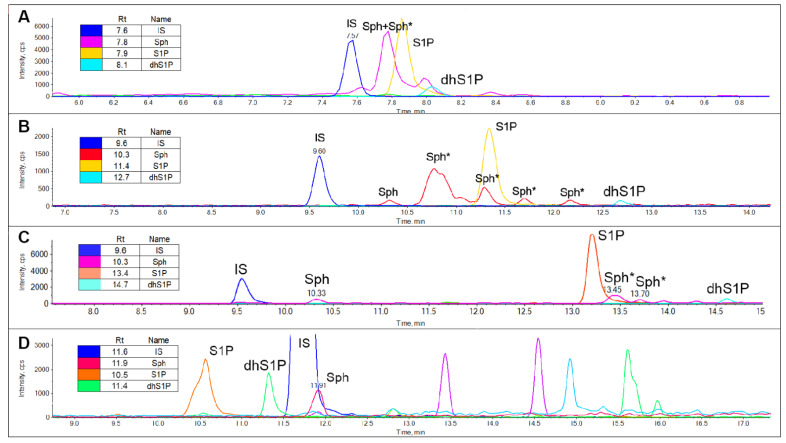
Chromatograms of plasma-free sphingoid bases analyzed by (**A**) Acquity BEH C18 with a short chromatography elution program, (**B**) Acquity BEH C18 with a long chromatography elution program, (**C**) Cortecs C18 with a long chromatography elution program and (**D**) Acquity BEH C18 with a long chromatography elution program after chemical derivatization with phenylisothiocyanate. In each panel * indicates the interferences on sphingosine transition. dhSph is not always appreciable since its concentration is markedly lower than other sphingosine bases. See Materials and Methods for the detailed LC-MS/MS conditions.

One of the main issues in the analysis of Sph and other sphingoid bases is that their levels in plasma and other biological matrices are not always high enough to allow a precise quantitation. Bearing in mind that the concentrations of sphingoid bases in human plasma range from 0.006 µM to 1.56 µM [19,20,21,22,23,24,25,26,27] (Supplementary Figure S1), it is critical to be aware of any possible interferents in the analysis. As particularly appraisable in Figure 3, choosing the appropriate column and chromatography conditions can make a huge difference in sphingoid bases analysis. In fact, many interfering signals of Sph are detected along with the chromatogram. While a short chromatography (Figure 3A) may seem an optimal choice for the analysis of sphingoid bases, the interferences over Sph are not even detected, and lengthening the runtime (Figure 3B), on the other hand, does not allow a clear distinction of Sph from its interfering signals. We resolved this issue by switching the column from an Acquity BEH C18 to a Cortecs C18; in fact, while Sph-interfering signals are still detected, they are completely separated from Sph (approximately three minutes apart), allowing an as close to reality as possible quantitation. Moreover, the use of a relative long elution program also enabled a sensible reduction of carry-over of phosphate derivatives, which can be displayed in run times inferior than 10 min.

#### 2.2.1. Sphingoid Bases Derivatization

In order to fix the issue of Sph-interfering signals, we evaluated whether a derivatization of the extract could be determined. As displayed in Figure 3D, the interfering signals of Sph completely disappeared and the chromatographic separation was excellent for all analytes. The detection of derivatives of Sph and dhSph is increased by the mean of 1.5–2.5-fold (Supplementary Table S1). On the other hand, though, the signal intensity of S1P is reduced by approximately 50%, which interferes with the intent of detecting sphingoid bases even in matrices and systems that may not be particularly enriched in these species (Supplementary Table S1). However, their quantification in plasma—which maintains a relatively high sphingoid bases concentration—can be achieved undeniably by either derivatizing their amine function or not, as displayed in Supplementary Figure S2. In the analytes considered here, the derivatization indeed unveiled slightly higher concentrations vs. the same samples not derivatized.

### 2.3. Performance in Human Plasma and Red Blood Cells

**Figure 4:**
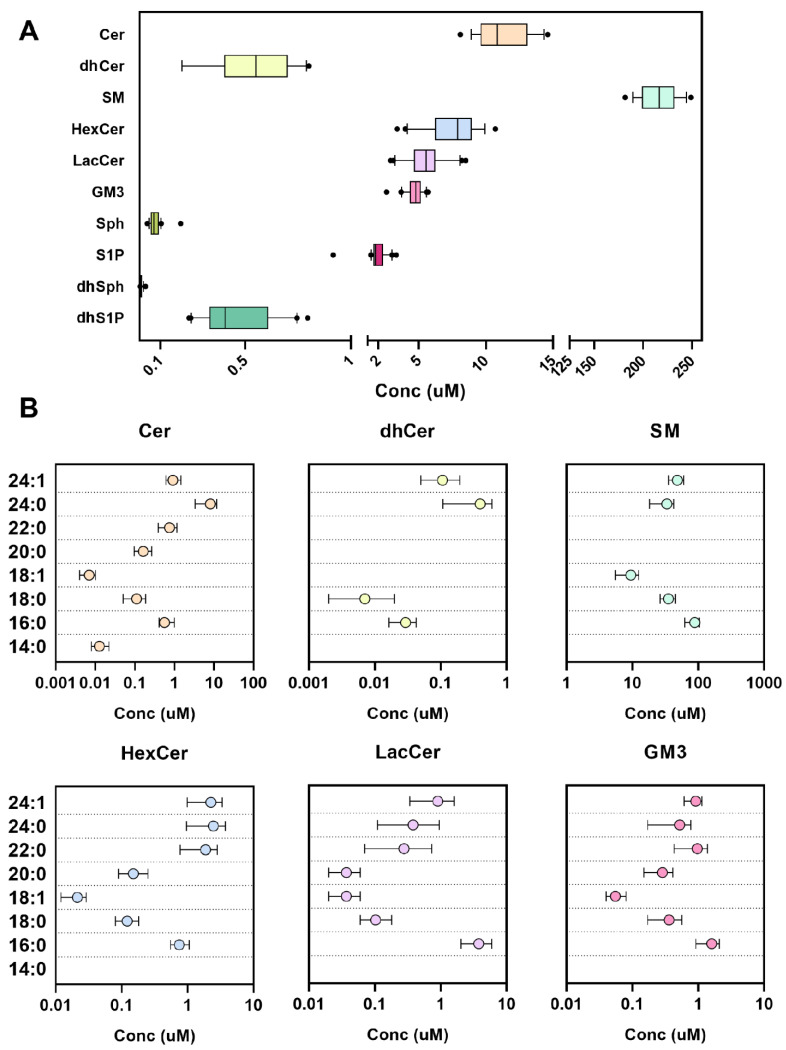
Plasma sphingolipids concentration in healthy volunteers (*n* = 20). (**A**) Concentrations of complex sphingolipids and free sphingoid bases (min–max, line at mean, dots represent the 10–90th percentile) as the sum of the species in each class and (**B**) divided according to their fatty acid composition (mean ± SD).

**Figure 5:**
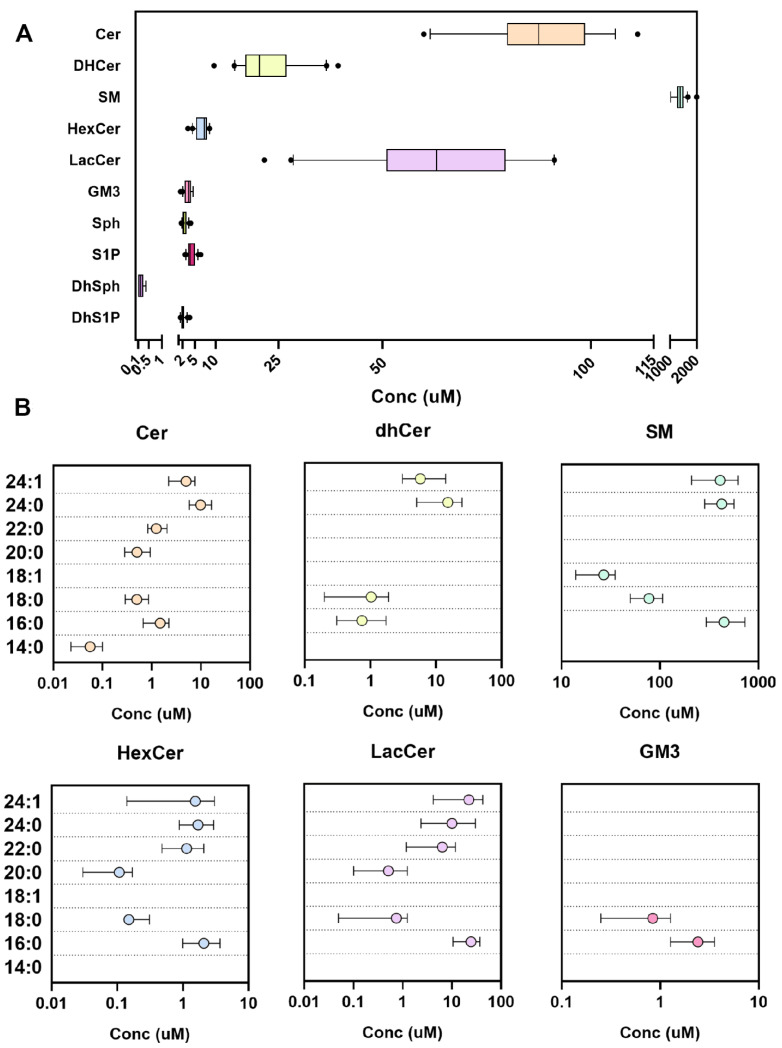
RBCs sphingolipid concentration in healthy volunteers (*n* = 20). (**A**) Concentrations of complex sphingolipids and free sphingoid bases (min–max, line at mean, dots represent the 10–90th percentile) as the sum of the species in each class and (**B**) divided according to their fatty acid composition (mean ± SD).

When we adopted the final extraction protocol (Materials and Methods, Section 3.4, protocol 4), for both complex sphingolipids and sphingoid bases (long chromatography on Cortecs C18), the concentration range of the analytes fell perfectly into those described in the literature [19,20,21,22,23,24,25,26,27] and the reproducibility of the methods was validated (Table 1 and Table 2). In Figure 4, the attained ranges are shown. In Table 3 and Table 4, furthermore, the results are expressed in numerical form and the percentage of analyzed species. Red blood cells (RBCs) sphingolipid concentrations [28,29,30,31,32] are introduced in Figure 5. The main sphingolipid in RBCs remains SM (87.5%) and Cer (5.8%) but with respect to the glycosphingolipids, lactosylceramides (LacCer) are prevalent (4% RBCs vs. 2.2% plasma), whereas in plasma the mono HexCer are predominant (3.0% plasma vs. 0.4% RBCs). The low-abundant dhCer are fairly detectable in plasma, accounting for less than 0.2% of total sphingolipids, but contrarily, in RBCs, they are more abundant, estimated at 1.4%. In Supplementary Table S2, the concentrations of sphingolipids in RBCs are reported in pmol/10^6^ cells.

## 3. Materials and Methods

### 3.1. Biological Samples from Healthy Volunteers

All subjects, who voluntarily agreed to participate in the study, were informed and authorization was obtained by signing a letter of consent. These participants were selected from a wider clinical trial that was approved by the institutional local ethical committee (Ospedale San Paolo, Milano, Italy). Blood from twenty volunteers was collected in the fasting state using K_2_EDTA as an anticoagulant, and the resulting plasma was obtained by centrifugation for 15 min at 1400× *g*. The recruited volunteers ranged in age from 18 to 85 and they were not diagnosed for cardiometabolic, liver or kidney diseases. Each volunteer was tested for complete blood count and their results had to fall within the medical laboratory’s physiological parameters in order to be included in the research. Prior to the analysis, plasma and RBCs were stored at −80 °C. All the procedures adopted in the present study were respectful of the ethical standards in the Helsinki Declaration. In order to study the method performances (Table 2), the implementations of different extraction protocols (Figure 1) and the effects of time and temperature on the recovery of sphingolipids (Figure 2), a pool of all the plasma and RBCs gathered (*n* = 20) was made and stored or processed as other samples. Otherwise, the use of individual samples was applied in the study of the sphingolipids’ physiological range in the biological matrix (Figure 4 and Figure 5, Table 3 and Table 4).

### 3.2. Chemicals and Reagents

The chemicals methanol, chloroform, formic acid, acetic acid, ammonium acetate, ammonium formate, dibutylhydroxytoluene (BHT), phenylisothiocyanate (PITC) and 4-nitrophenylisothiocyanate (NO_2_PITC) were all at analytical grade and were purchased from Sigma-Aldrich (St. Louis, MO, USA). All aqueous solutions were prepared using purified water at a Milli-Q grade (Burlington, MA, USA). Lipid standards were purchased from Avanti Polar (supplied by Sigma-Aldrich, St. Louis, MO, USA).

### 3.3. LC-MS/MS

The LC-MS/MS consisted of an LC Dionex 3000 UltiMate (ThermoFisher Scientific, Waltham, MA, USA) coupled to a tandem mass spectrometer AB Sciex 3200 QTRAP (AB Sciex, Concord, ON, Canada) equipped with electrospray ionization TurboIonSpray™ source operating in positive mode (ESI+).

#### 3.3.1. Sphingolipids and Glycosphingolipids

The instrument parameters were: CUR 25, GS1 45, GS2 50, capillary voltage 5.5 kV and source temperature 300 °C. Spectra were acquired by multiple reaction monitoring, scanning for each analyte, the transitions reported in Supplementary Table S3. To chromatographically isolate the analytes, we used a reverse-phase Acquity BEH C8 column 1.7 μm, 2.1 × 100 mm (Waters, Milford, MA, USA) equipped with pre-column, using as mobile phases (A) water + 0.2% formic acid + 2 mM ammonium formate and (B) methanol + 0.2% formic acid + 1 mM ammonium formate. The flow rate was 0.3 mL/min and the column temperature was set to 30 °C. The elution gradient (%B) was set as follows: 0–3 min (80–90%), 3.0–6.0 min (90%), 6.0–19.0 min (90–99%), 19.0–20.0 min (99–80%), held until 24 min. Five microliters of clear supernatant were directly injected into LC-MS/MS. Due to the lack of authentic standards for every fatty acid chain, those which are not available were quantified as a reference of the closest sphingolipids subspecies.

#### 3.3.2. Free Sphingoid Bases

The instrument parameters were: CUR 25, GS1 45, GS2 55, capillary voltage 5.5 kV and source temperature 500 °C. Spectra were acquired by multiple reaction monitoring, scanning for each analyte, the transitions reported in Supplementary Table S4. Two columns were tested: reverse-phase Acquity BEH C18 column 1.7 μm, 2.1 × 100 mm (Waters, MA, USA) and reverse-phase Cortecs C18 1.6 μm, 2.1 × 100 mm (Waters, MA, USA). Both columns were equipped with pre-column and the mobile phase was (A) water + 0.2% formic acid + 2 mM ammonium formate and (B) methanol + 0.2% formic acid + 1 mM ammonium formate.

*Short chromatography (BEH C18).* The elution gradient (%B) was set as follows: 0–2 min (20%), 2–4 min (20–99%), 4–7 min (99%), 7–7.5 min (99–20%), held until 10 min [33]. The flow rate was 0.3 mL/min and the column temperature was set to 30 °C.

*Long chromatography (BEH C18).* The elution gradient (%B) was set as follows: 0–12 min (70–85%), 12.0–12.2 min (85–99%), 12.2–15.0 min (99%), 15.0–15.2 min (99–70%), held until 20 min. Five microliters of clear aqueous supernatant were directly injected into LC-MS/MS. The flow rate was 0.3 mL/min and the column temperature was set to 30 °C.

*Long chromatography (Cortecs C18).* The elution gradient (%B) was set as follows: 0–12 min (70–85%), 12.0–12.2 min (85–99%), 12.2–15.0 min (99%), 15.0–15.2 min (99–70%), held until 20 min. Three microliters of clear aqueous supernatant were directly injected into LC-MS/MS. The flow rate was 0.2 mL/min and the column temperature was set to 30 °C.

#### 3.3.3. Sphingoid Bases as Phenylthiourea Derivatives

The instrument parameters were: CUR 25, GS1 45, GS2 55, capillary voltage 5.5 kV and source temperature 500 °C. Spectra were acquired by multiple reaction monitoring, scanning for each PITC or NO_2_PITC derivative using the transitions reported in Supplementary Tables S5 and S6, respectively. To chromatographically isolate the analytes, we used a reverse-phase Cortecs C18 1.6 μm, 2.1 × 100 mm (Waters, MA, USA) equipped with pre-column using as mobile phase (A) water + 0.2% formic acid + 2 mM ammonium formate and (B) methanol + 0.2% formic acid + 1 mM ammonium formate. The flow rate was 0.3 mL/min and the column temperature was 40 °C. The elution gradient (%B) was set as below: 0–16.0 min (70–99%), 16.0–17.0 min (99%), 17.0–17.2 min (99–70%), held until 20 min. Three microliters of clear supernatant were directly injected into LC-MS/MS.

### 3.4. Extraction Procedures

**Protocol 1.** Plasma (25 µL) was diluted with water (75 µL) before being mixed with a methanol/chloroform solution (850 µL, 2:1, *v*/*v*). The lipids were extracted by ice-sonication and thermo-shaking (1 h, 1000 rpm, rt) of the plasma samples. The organic phase was separated via centrifugation (15 min at 20,000× *g*) and evaporated under a stream of nitrogen. The residues were dissolved in 100 µL of methanol + 0.1 mM BHT and withdrawn in a glass vial.

**Protocol 2.** Plasma (25 µL) was diluted with water (75 µL) before being mixed with a methanol/chloroform solution (850 µL, 2:1, *v*/*v*). The lipids were extracted by ice-sonication and thermo-shaking (overnight, 1000 rpm, 48 °C) of the plasma samples. The organic phase was separated via centrifugation (15 min at 20,000× *g*) and evaporated under a stream of nitrogen. The residues were dissolved in 100 µL of methanol + 0.1 mM BHT and withdrawn in a glass vial.

**Protocol 3.** Plasma (25 µL) was diluted with water (75 µL) before being mixed with a methanol/chloroform solution (850 µL, 2:1, *v*/*v*). The lipids were extracted by ice-sonication and thermo-shaking (1 h, 1000 rpm, rt) of the plasma samples. They went through alkaline methanolysis (75 µL KOH 1M, 2 h at 38 °C) and were then neutralized by the addition of glacial acetic acid (4 µL). The organic phase was separated via centrifugation (15 min at 20,000× *g*) and evaporated under a stream of nitrogen. The residues were dissolved in 100 µL of methanol + 0.1 mM BHT and were withdrawn in a glass vial.

**Protocol 4.** Plasma (25 µL) was diluted with water (75 µL) and added with a methanol/chloroform mixture (850 µL, 2:1, *v*/*v*). The lipids were extracted by ice-sonication and thermo-shaking (overnight, 1000 rpm, 48 °C) of the plasma samples. They went through alkaline methanolysis (75 µL KOH 1M, 2 h at 38 °C) and were then neutralized by the addition of glacial acetic acid (4 µL). The organic phase was separated via centrifugation (15 min at 20,000× *g*) and evaporated under a stream of nitrogen. The residues were dissolved in 100 µL of methanol + 0.1 mM BHT and were withdrawn in a glass vial.

### 3.5. Derivatization of Free Sphingoid Bases

The amine group reacted with PITC to mainly produce the phenylthiourea [34] derivatives of sphingoid bases. An aliquot of the final extract (25 µL) was withdrawn into a new glass vial and PITC derivatization was performed by adding a solution of PITC/pyridine (25 µL, 100 mM PITC in methanol/pyridine 1:1, *v*/*v*). The vial was capped and heated at 80 °C for 1 h. Prior to analysis, pure formic acid (5 µL) was added. The best conditions for derivatization were investigated as reported in Supplementary Table S7. NO_2_PITC derivatives were obtained with the same protocol, adding to the final extract (25 µL) a solution of NO_2_PITC/pyridine (25 µL, 100 mM NO_2_PITC in methanol/pyridine 1:1, *v*/*v*). Supplementary Tables S5 and S6 report the mass spectrometry conditions for PITC and NO_2_PITC derivatives.

### 3.6. Condition for Alkaline Methanolysis

The recovery of low abundant sphingolipids is commonly accomplished through alkaline methanolysis which causes the lysis of the ester linkage while retaining the intact amide bond. The percentage of intact phospholipids was used to monitor the reaction over time (1, 2, 6, 12 h). The instrument parameters were: CUR 25, GS1 40, GS2 45, capillary voltage 5.5 kV and source temperature 400 °C. Spectra were acquired by multiple reaction monitoring, scanning for DPPC (m/z 734.6 > 184.1) and the internal standard PC d7 (m/z 753.6 > 184.1). To chromatographically isolate the analytes, we used a reverse-phase Acquity BEH C8 column 1.7 μm, 2.1 × 100 mm (Waters, MA, USA) equipped with pre-column, using as mobile phases (A) water + 0.2% formic acid + 2 mM ammonium formate and (B) methanol + 0.2% formic acid + 1 mM ammonium formate. The flow rate was 0.3 mL/min and the column temperature was 35 °C. The elution gradient (%B) was set as below: 0–14 min (80–99%), 14–20 min (99%), 20–20.1 min (99–80%), held until 25 min. Five microliters of clear supernatant were directly injected into LC-MS/MS.

### 3.7. Comparison between Traditional Biphasic and Monophasic Extractions

The performances of the operating protocol described in Section 3.6 were juxtaposed with the micro-scaled versions of the classical liquid–liquid extraction protocols first proposed by Folch and Bligh-Dyer [15,16]. The comparison between the three extraction protocols (Folch, Bligh-Dyer and monophase extraction) was assessed in triplicate using the same plasma pool, obtained by combining a suitable amount of each individual sample (*n* = 20).

### 3.8. Time and Temperature for Isolating Sphingolipids from a Biological Matrix

The same plasma pool, already mentioned above (25 µL), was diluted with water (75 µL) before being mixed with a methanol/chloroform solution (850 µL, 2:1, *v*/*v*); it was ice-sonicated and extracted by following this scheme: (1) ambient temperature extraction (22 °C) for either 1/2/4 or 12 h; (2) cold extraction (4 °C) for either 1/2/4 or 12 h; (3) warm extraction (38 °C) for either 1/2/4 or 12 h; (4) hot extraction (48 °C) for either 1/2/4 or 12 h. Then, the samples went through alkaline methanolysis (75 µL KOH 1M, 2 h at 38 °C) and were then neutralized by the addition of glacial acetic acid (4 µL). The organic phase was separated via centrifugation (15 min at 20,000× *g*) and evaporated under a stream of nitrogen. The residues were dissolved in 100 µL of methanol + 0.1 mM BHT and withdrawn in a glass vial.

### 3.9. Operating Protocol for Plasma Samples

Plasma (25 µL) was diluted with water (75 µL) before being mixed with a methanol/chloroform solution (850 µL, 2:1, *v*/*v*). The lipids were extracted by ice-sonication and thermo-shaking (1 h, 1000 rpm, 38 °C) of the plasma samples. They went through alkaline methanolysis (75 µL KOH 1M, 2 h at 38 °C) and were then neutralized by the addition of glacial acetic acid (4 µL). The organic phase was separated via centrifugation (15 min at 20,000× *g*) and evaporated under a stream of nitrogen. The residues were dissolved in 100 µL of methanol + 0.1 mM BHT and withdrawn in a glass vial.

### 3.10. Red Blood Cells Protocol

RBCs (10 µL) were lysed by hypotonic shock in double-distilled water (490 µL). An aliquot of the lysed solution (25 µL, which on average corresponds to 2.5 × 10^6^ cells or 0.5 µL of the initial sample) was diluted with water (75 µL) before being mixed with a methanol/chloroform solution (850 µL, 2:1, *v*/*v*). The lipids were extracted by ice-sonication and thermo-shaking (1 h, 1000 rpm, 38 °C). They went through alkaline methanolysis (75 µL KOH 1M, 2 h at 38 °C) and were then neutralized by the addition of glacial acetic acid (4 µL). The organic phase was separated via centrifugation (15 min at 20,000× *g*) and evaporated under a stream of nitrogen. The residues were dissolved in 100 µL of methanol + 0.1 mM BHT and withdrawn in a glass vial.

### 3.11. Methods Performances

The methods performances were tested using the same plasma pool obtained by combining suitable amounts of each sample (*n* = 20). The precision of the methods was calculated as the coefficient of variation (CV%) by extracting five times the same pool sample in a day (intra-day) and another five times the day after (inter-day).

### 3.12. Statistical Analysis

The software used for the visualization of the results and the univariate statistical analysis was GraphPad Prism 9.0 (GraphPad Software, Inc., La Jolla, California, USA). For repeated measures comparison among different groups, repeated measured one-way ANOVA with Dunnett post hoc test was performed. In all tests, *p* < 0.05 was considered statistically significant.

## 4. Conclusions

In this work, we assessed whether different extraction and analytical protocols could affect the results attained from a targeted sphingolipidomics analysis. The single-phase extraction followed by an alkaline methanolysis seems to be crucial for acquiring as accurate as possible results, while its duration and temperature might not be as significant. Another pivotal aspect in the analyses of sphingolipids is represented by the choice of appropriate columns for distinctively analyzing complex sphingolipids and sphingoid bases. On the other hand, derivatization of the sphingoid bases, while effective on paper, especially on non-phosphorylated species, does not allow a consistent improvement for the analysis of phosphorylated sphingoid bases. For this purpose, the use of a proper column, in this case a Cortecs C18, coupled with a full-length chromatography, seems to be much more convenient, in order to efficiently separate Sph from its interfering peaks and still appreciate all other sphingoid bases.

## Figures and Tables

**Table 1:** Intra- (*n* = 5 independent extraction replicates) and inter-days (*n* = 10 independent extraction replicates) precision for the analysis of the whole panel of plasma sphingolipids on a plasma pool from healthy volunteers (*n* = 20). The analyses of the sphingolipids and sphingoid bases were performed as described in Section 3.3.1 and Section 3.3.2 (long chromatography), respectively.

Class	CV% Intra-Day (*n* = 5)	CV% Inter-Days (*n* = 10)
Cer	1.6	7.9
dhCer	3.8	7.6
SM	3.8	7.9
HexCer	7.2	6.0
LacCer	7.8	12.8
GM3	7.6	14.0
Sph	4.2 (4.8)	4.8 (13.1)
S1P	5.0 (2.1)	3.8 (12.4)
dhSph	10.1 (11.1)	11.2 (14.8)
dhS1P	8.1 (2.4)	6.8 (11.9)

**Table 2:** Intra- (*n* = 5 independent extraction replicates) and inter-days (*n* = 10 independent extraction replicates) precision for the analysis of the whole panel of RBCs sphingolipids on an RBCs pool from healthy volunteers (*n* = 20). The analyses of the sphingolipids and sphingoid bases were performed as described in Section 3.3.1 and Section 3.3.2 (long chromatography), respectively.

Class	CV% Intra-Day (*n* = 5)	CV% Inter-Days (*n* = 10)
Cer	7.4	10.5
dhCer	7.2	15.0
SM	8.4	9.3
HexCer	13.4	10.5
LacCer	12.4	9.8
GM3	11.2	11.3
Sph	9.6	13.9
S1P	9.4	8.3
dhSph	9.9	8.0
dhS1P	7.7	10.0

**Table 3:** Plasma EDTA sphingolipids levels (μM) in healthy volunteers (*n* = 20) expressed as min–max, mean ± SD and percentage over total sphingolipid content.

Conc (µM)	Min	Max	Mean ± SD (*n* = 20)	%
Cer	8.1	14.6	11.2 ± 2.0	4.5
dhCer	0.2	0.8	0.54 ± 0.20	0.2
SM	181.7	248.9	217.9 ± 19.3	87.2
HexCer	3.4	10.7	7.6 ± 1.9	3.0
LacCer	2.9	8.5	5.5 ± 1.4	2.2
GM3	2.6	5.7	4.7 ± 0.7	1.9
Sph	0.04	0.2	0.07 ± 0.034	0.03
S1P	0.9	3.3	2.0 ± 0.593	0.8
dhSph	0.003	0.03	0.01 ± 0.006	0.004
dhS1P	0.23	0.80	0.45 ± 0.177	0.2

**Table 4:** RBCs sphingolipid levels (μM) in healthy volunteers (*n* = 20) expressed as min–max, mean ± SD and percentage over total sphingolipid content.

Conc (µM)	Min	Max	Mean ± SD (*n* = 20)	%
Cer	59.9	148.5	92.8 ± 21.1	5.9
dhCer	9.6	39.3	22.7 ± 8.0	1.4
SM	854.6	2000	1387 ± 237.3	87.4
HexCer	3.3	8.5	6.7 ± 1.5	0.4
LacCer	21.6	115.9	64.2 ± 23.8	4.0
GM3	1.5	4.6	3.3 ± 0.9	0.2
Sph	1.7	4.0	2.5 ± 0.64	0.1
S1P	2.6	6.4	4.1 ± 1.041	0.3
dhSph	0.1	0.4	0.2 ± 0.112	0.01
dhS1P	1.5	3.7	2.2 ± 0.563	0.2

## Data Availability

The data presented in this study are available in article and Supplementary Materials.

## Supplementary Materials

The following supporting information can be downloaded at: https://www.mdpi.com/article/10.3390/metabo12050450/s1. Figure S1: Sphingoid bases concentrations in human plasma, according to scientific literature [18,19,20,21,22,23,24,25,26] (see paper for the references), Figure S2: Comparison between the sphingoid bases concentrations evidenced with (D, *n* = 20) or without (ND, *n* = 20) derivatization with phenylisothiocyanate, Table S1: Differences in signal intensities, expressed as fold-change on underivatized analytes, between the same concentration of sphingoid bases (1 µM) after derivatization, Table S2: RBCs sphingolipids levels (pmol/10^6^ cells) in healthy volunteers (*n* = 20) were expressed as min–max and mean ± SD, Table S3: Mass spectrometry parameters for the analysis of complex sphingolipids. In bold are reported the internal standards (IS) used for each package of lipids, Table S4: Mass spectrometry parameters for the analysis of free sphingoid bases, Table S5: Mass spectrometry parameters for the analysis of sphingoid bases as phenylthiourea derivatives after reaction with phenylisothiocyanate, Table S6: Mass spectrometry parameters for the analysis of sphingoid bases as nitrophenylthiourea derivatives after reaction with 4-nitrophenylisothiocyanate, Table S7: The yield of derivatization products after different times and temperatures of reaction. Each experiment was conducted by adding the same amount of reagents, derivatizing agent (PITC) and catalyzer (Pyridine).

## Abbreviations

BHT, dibutylhydroxytoluene; Cer, ceramides; dhCer, dihydroceramides; dhS1P, dihydrosphingosine-1-phosphate; dhSpH, dihydrosphingosine; DPPC, dipalmitoylphosphatidylcholine; GM3, gangliosides; HexCer, hexosylceramides; LacCer, lactosylceramides; NO2PITC, 4-nitrophenylisothiocyanate; PC, phosphatidylcholine; PC d7, phosphatidylcholine (15:0–18:1) d7; PITC, phenylisothiocyanate; RBC, red blood cell; S1P, sphingosine-1-phosphate; Sph, sphingosine; SM, sphingomyelins.
