# Supplementary material for: An Update on Sphingolipidomics: Is Something Still Missing? Some Considerations on the Analysis of Complex Sphingolipids and Free-Sphingoid Bases in Plasma and Red Blood Cells
Source: Metabolites. 2022 May 17;12(5):450. doi: 10.3390/metabo12050450 (PMC9147510; doi:10.3390/metabo12050450)
Supplement: Supplementary file 1 [file metabolites-12-00450-s001.zip › metabolites-1730678-supplementary.pdf]

metabolites

Supplementary materials

An Update on Sphingolipidomics: Is Something Still Missing? Some Consideration on the Analysis of Complex Sphingolipids and Free-Sphingoid Bases in Plasma and Red Blood Cells

Camillo Morano[1], Aida Zulueta[2], Anna Caretti[3], Gabriella Roda[1], Rita Paroni[3], Michele Dei Cas[3,*]

[1] Department of Pharmaceutical Sciences, Università degli Studi di Milano, Milan, Italy; camillo.morano@unimi.it; gabriella.roda@unimi.it
[2] Neurorehabilitation Department, IRCCS Istituti Clinici Scientifici Maugeri, 20138 Milan, Italy; aida.zuluetamorales@icsmaugeri.it;
[3] Department of Health Sciences, Università degli Studi di Milano, Milan, Italy; anna.caretti@unimi.it rita.paroni@unimi.it; michele.deicas@unimi.it
* Correspondence: Dr. Michele Dei Cas, PhD, Department of Health Sciences, Università degli Studi di Milano, Milan, Italy, email address: michele.deicas@unimi.it

Supplementary materials legend:

1. **Supplementary Figure S1.** Sphingoid bases concentrations in human plasma, according to scientific literature [18–26], (see paper for the references).
2. **Supplementary Figure S2.** Comparison between the sphingoid bases concentrations evidenced with (D, n=20) or without (ND, n=20) derivatization with phenylisothiocyanate.
3. **Supplementary Table S1.** Differences in signal intensities, expressed as fold-change on underivatized analytes, between the same concentration of sphingoide bases (1 μM) after derivatization
4. **Supplementary Table S2.** RBCs sphingolipids levels (pmol/10^6 cells) in healthy volunteers (n=20) were expressed as min-max and mean±SD
5. **Supplementary Table S3.** Mass spectrometry parameters for the analysis of complex sphingolipids. In bold are reported the internal standards (IS) used for each package of lipids
6. **Supplementary Table S4**. Mass spectrometry parameters for the analysis of free sphingoid bases
7. **Supplementary Table S5.** Mass spectrometry parameters for the analysis of sphingoid bases as phenylthiourea derivatives after reaction with phenylisothiocyanate
8. **Supplementary Table S6.** Mass spectrometry parameters for the analysis of sphingoid bases as nitrophenylthiourea derivatives after reaction with 4-nitrophenylisothiocyanate
9. **Supplementary Table S7.** The yield of derivatization products after different times and temperatures of reaction. Each experiment was conducted by adding the same amount of reagents, derivatizing agent (PITC), and catalyzer (Pyridine)

Supplementary Figure S1. Sphingoid bases concentrations in human plamsa, according to scientific literature [18–26], (see paper for the references).

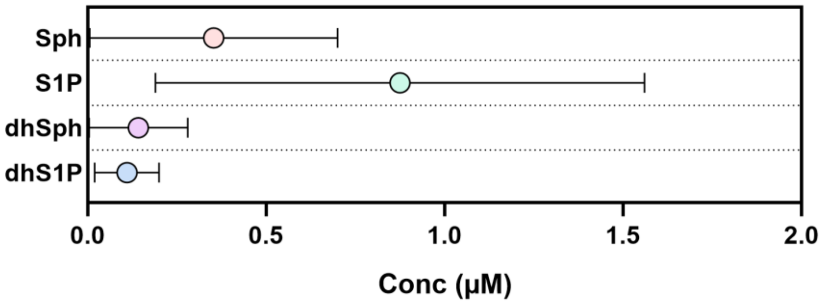

Supplementary Figure S2. Comparison between the sphingoid bases concentrations evidenced with (D, n=20) or without (ND, n=20) derivatization with phenylisothiocyanate.

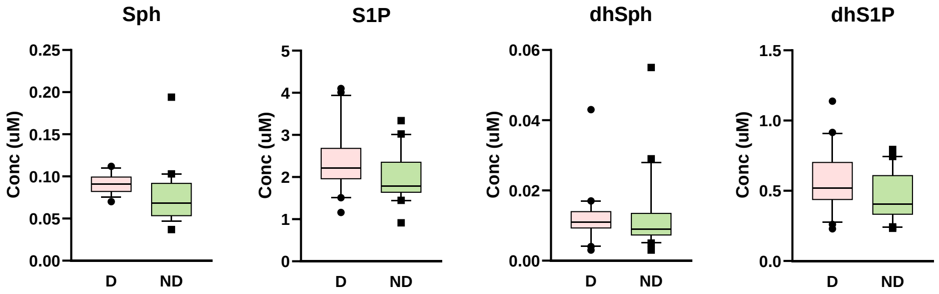

Supplementary Table S1. Differences in signal intensities, expressed as fold-change on underivatized analytes, between the same concentration of sphingoide bases (1 μM) after derivatization.

Sphingoid base	Free	PITC	NO_2PITC
3KS	1	3.75	3.00
Sph	1	1.56	1.66
S1P	1	0.50	0.34
dhSph	1	2.80	2.40
dhS1P	1	1.00	0.54

Supplementary Table S2. RBCs sphingolipids levels (pmol/10^6 cells) in healthy volunteers (n=20) were expressed as min-max and mean±SD.

Conc (μM)	Min	Max	Mean±SD (n=20)
Cer	12	29.7	18.6±4.2
dhCer	1.9	7.9	4.5±1.6
SM	171	400	277.4±47.5
HexCer	0.7	1.7	1.345±0.302
LacCer	4.3	23	12.83±4.765
GM3	0.3	0.9	0.655±0.173
Sph	0.34	2.3	0.698±0.55
S1P	0.52	3.4	1.11±0.795
dhSph	0.02	0.4	0.071±0.082
dhS1P	0.3	2.2	0.623±0.512

Supplementary Table S3. Mass spectrometry parameters for the analysis of complex sphingolipids. In bold are reported the internal standards (IS) used for each package of lipids.

Name	MRM (+)	DP (eV)	CE (eV)
Ceramide (Cer) 12:0 (IS)	482.7 > 264.4	40	30
Cer 14:0	510.7 > 264.4	40	33
Cer 16:0	538.8 > 264.4	40	36
Cer 18:1	564.8 > 264.4	40	35
Cer 18:0	566.8 > 264.4	40	36
Cer 20:0	594.8 > 264.4	40	38
Cer 22:0	622.9 > 264.4	40	39
Cer 24:1	648.9 > 264.4	40	42
Cer 24:0	650.9 > 264.4	40	33
Dihydroceramide (dhCer) 16:0	540.4 > 266.4	40	35
dhCer 18:1	566.5 > 266.4	40	35
dhCer 18:0	568.5 > 266.4	40	39
dhCer 24:1	650.5 > 266.4	40	40
dhCer 24:0	652.5 > 266.4	40	40
Sphingomyelin (SM) 12:0 (IS)	647.6 > 184.3	40	29
SM 16:0	703.5 > 184.1	60	40
SM 18:0	731.6 > 184.1	60	40
SM 18:1	729.6 > 184.1	60	40
SM 24:0	815.7 > 184.1	60	40
SM 24:1	813.7 > 184.1	60	40
Hexosylceramide (HexCer) 12:0 (IS)	644.5 > 264.3	40	50
HexCer 16:0	700.6 > 264.3	40	50
HexCer 18:0	728.6 > 264.3	40	50
HexCer 18:1	726.6 > 264.3	40	50
HexCer 20:0	756.6 > 264.3	40	50
HexCer 22:0	784.7 > 264.3	40	50
HexCer 24:0	812.7 > 264.3	40	50
HexCer 24:1	810.7 > 264.3	40	50
Lactosylceramide (LacCer) 16:0	862.6 > 264.3	60	60
LacCer 18:0	890.7 > 264.3	60	60
LacCer 18:1	888.7 > 264.3	60	60
LacCer 20:0	918.7 > 264.3	60	60
LacCer 22:0	946.7 > 264.3	60	60
LacCer 24:0	974.8 > 264.3	60	60
LacCer 24:1	972.7 > 264.3	60	60
Ganglioside GM3 (GM3) 16:0	1153.7 > 264.3	70	60
GM3 18:0	1181.7 > 264.3	70	60
GM3 18:1	1179.8 > 264.3	70	60
GM3 20:0	1209.8 > 264.3	70	60
GM3 22:0	1237.8 > 264.3	70	60
GM3 24:0	1265.8 > 264.3	70	60
GM3 24:1	1263.8 > 264.3	70	60

Supplementary Table S4. Mass spectrometry parameters for the analysis of free sphingoid bases.

Name	MRM (+)	DP (eV)	CE (eV)
Sphinganine d17:0 (IS)	288.4 > 252.0	21	20
3-ketosphinganine (3KS)	300.0 > 270.4	46	23
Sphingosine (Sph)	300.3 > 282.2	21	17
Sphinosine-1-phosphate (S1P)	380.2 > 264.3	26	21
Dihydrosphingosine (dhSph)	302.2 > 284.5	31	19
Dihydrosphingosine-1- phosphate (dhS1P)	382.4 > 284.5	46	19

Supplementary Table S5. Mass spectrometry parameters for the analysis of sphingoid bases as phenylthiourea derivatives after reaction with phenylisothiocyanate.

Name	MRM (+)	DP (eV)	CE (eV)
Sphinganine d17:0 (IS)	423.3 > 270.0	50	27
3KS	435.3 > 282.2	46	29
Sph	435.3 > 399.5	36	21
S1P	515.3 > 399.5	50	31
dhSph	437.3 > 419.3	50	25
dhS1P	517.3 > 419.3	51	23

Supplementary Table S6. Mass spectrometry parameters for the analysis of sphingoid bases as nitrophenylthiourea derivatives after reaction with 4-nitrophenylisothiocyanate.

Name	MRM (-)	DP (eV)	CE (eV)
Sphinganine d17:0 (IS)	466.2>137.0	-50	-45
3KS	478.3>137.0	-50	-45
Sph	478.3>137.0	-50	-45
S1P	588.2>378.2	-70	-28
dhSph	480.3>137.0	-50	-45
dhS1P	560.3>380.2	-70	-50

Supplementary Table S7. The yield of derivatization products after different times and temperatures of reaction. Each experiment was conducted by adding the same amount of reagents, derivatizing agent (PITC) and catalyzer (Pyridine).

Temperature (°C)	***40***	***60***	***60***	***70***	***70***	***80***
Time (min)	***15***	***60***	***120***	***60***	***120***	***60***
Sphinganine d17:0 (IS)	99.9	100.0	100.0	100.0	100.0	100.0
3KS	99.7	100.0	100.0	100.0	100.0	100.0
Sph	97.6	99.8	99.9	100.0	100.0	99.9
S1P	8.4	77.5	90.0	97.4	100.0	99.4
dhSph	97.7	99.9	99.9	99.9	99.9	100.0
dhS1P	28.8	92.4	96.9	99.2	100.0	99.8
